# Chlorinated Auxins—How Does *Arabidopsis Thaliana* Deal with Them?

**DOI:** 10.3390/ijms21072567

**Published:** 2020-04-07

**Authors:** Antje Walter, Lorenzo Caputi, Sarah O’Connor, Karl-Heinz van Pée, Jutta Ludwig-Müller

**Affiliations:** 1 Institute of Botany, Technische Universität Dresden, 01062 Dresden, Germany; ant-wa@web.de; 2 Department of Natural Product Synthesis, Max Planck Institute for Chemical Ecology, 07745 Jena, Germany; lcaputi@ice.mpg.de (L.C.); oconnor@ice.mpg.de (S.O.); 3 Faculty of Chemistry and Food Chemistry, Technische Universität Dresden, 01062 Dresden, Germany; karl-heinz.van_pee@tu-dresden.de

**Keywords:** *Arabidopsis thaliana*, auxin, bacterial halogenase, chloro-tryptophan, chloro-indole acetic acid, chloro-indole acetonitrile, plant natural products, plant metabolic engineering

## Abstract

Plant hormones have various functions in plants and play crucial roles in all developmental and differentiation stages. Auxins constitute one of the most important groups with the major representative indole-3-acetic acid (IAA). A halogenated derivate of IAA, 4-chloro-indole-3-acetic acid (4-Cl-IAA), has previously been identified in *Pisum sativum* and other legumes. While the enzymes responsible for the halogenation of compounds in bacteria and fungi are well studied, the metabolic pathways leading to the production of 4-Cl-IAA in plants, especially the halogenating reaction, are still unknown. Therefore, bacterial flavin-dependent tryptophan-halogenase genes were transformed into the model organism *Arabidopsis thaliana*. The type of chlorinated indole derivatives that could be expected was determined by incubating wild type *A. thaliana* with different Cl-tryptophan derivatives. We showed that, in addition to chlorinated IAA, chlorinated IAA conjugates were synthesized. Concomitantly, we found that an auxin conjugate synthetase (GH3.3 protein) from *A. thaliana* was able to convert chlorinated IAAs to amino acid conjugates in vitro. In addition, we showed that the production of halogenated tryptophan (Trp), indole-3-acetonitrile (IAN) and IAA is possible in transgenic *A. thaliana* in planta with the help of the bacterial halogenating enzymes. Furthermore, it was investigated if there is an effect (i) of exogenously applied Cl-IAA and Cl-Trp and (ii) of endogenously chlorinated substances on the growth phenotype of the plants.

## 1. Introduction

The halogenated auxin 4-chloro-indole-3-acetic acid (4-Cl-IAA) is among the strongest auxins identified in plants up to date [1]. It stimulates, amongst other activities, pericarp growth and rooting in pea [1], and corn coleoptile extension [2]. The 4-Cl-IAA concentration required compared to the non-chlorinated auxin indole-3-acetic acid (IAA) is ten times lower in order to achieve the same effects [3]. A reason for this could be the increased stability of the chlorinated compound [4]. In pea, it is assumed that 4-Cl-IAA is transported by the young seeds into the pods, where it subsequently ensures its growth [1]. Furthermore, several studies show a close association of 4-Cl-IAA to other hormones. For example, on the one hand, 4-Cl-IAA stimulates certain genes in gibberellin [5] and ethylene synthesis [6], but on the other hand, chlorinated auxin represses genes for gibberellin catabolism [5].

Evidence for the occurrence of 4-Cl-IAA was first found in germinating seeds of *Pisum sativum* (pea) [7,8] by detecting its methyl ester. Since that time, the free acid was also found [9,10] and the compound has been identified in other plants [1,11,12,13]. In peas 4-Cl-IAA was detected in developing seeds [9], surface plant parts, roots and cotyledons of three-day-old etiolated seedlings [10] and fruits [14]. There are different studies about the occurrence and endogenous concentration of 4-Cl-IAA in other plant species. Pless et al. [15] found the auxin in fully developed leaves of broad beans in high concentrations, whereas in recent studies only small concentrations could be detected, e.g., in young and ripe seeds of beans, respectively [16]. Chlorinated IAA has also been described in other leguminous plants, such as *Medicago truncatula* and *Trifolium repens* [16]. However, little is known about the synthesis of this chlorinated auxin itself, especially on enzymes catalyzing the halogenating reaction.

Halogenated compounds occur widely in nature. Today more than 5000 naturally produced halogenated substances are known and their number is increasing [17]. The structures range from quite simple compounds such as methyl bromide or bromomethane [18] to highly complex polyketides and oligopeptides such as antibiotics like rebeccamycin [19]. Their functions can be very diverse and range from antimicrobial activities [20,21] to the plant growth substance thienodolin [22]. Many halogenated substances have been found in fungi and bacteria especially. The biosynthetic routes of these compounds and the enzymes involved are were elucidated in many cases. One class of halogenating enzymes are bacterial flavin-dependent halogenases, which can halogenate the amino acid tryptophan regioselectively at defined positions of the indole ring [23,24]. Besides tryptophan (Trp), some of these halogenases can also use IAA as a substrate in vitro [25]. However, the biosynthetic pathway for 4-Cl-IAA in plants is up to now not well understood. The majority of IAA in plants is derived from Trp as a precursor via different possible pathways [26]. For peas it was shown that 4-Cl-IAA originates from the chlorinated amino acid 4-chloro-tryptophan (4-Cl-Trp) via the indole-3-pyruvic acid pathway [27]. So far, no precursor of IAA, i.e., Trp, has been detected in a chlorinated version [16]. Consequently, there are no molecular genetic studies on the synthesis of Cl-Trp and Cl-IAA in plants.

To show the potential of chlorinated compounds synthesized by plants, different strategies have been used. Employing *Catharanthus roseus* hairy roots the existence of new halogenated compounds with modified properties after the incorporation of simple halogenated precursor was shown [28]. More recently, either original bacterial 5-Cl-Trp halogenase genes or codon optimized genes were generated and introduced under the control of the 35S cauliflower mosaic virus (CaMV) promoter into *Arabidopsis thaliana*, and it was shown that these plants contained chlorinated Trps as well as IAA [25]. In this work, we have extended these studies to include the genes of two additional regioselective Trp halogenases. We have analyzed the effect of the expression of three different halogenase genes encoding regioselective enzymes for the 5-, 6- and 7-position of the indole ring in *A. thaliana*. Furthermore, we have evaluated the capacity of these transgenic lines to produce chlorinated Trp and other indole derivatives. In addition, wild type plants were incubated with different halogenated compounds to test the effect of the substances on plant growth. Such investigations have so far not systematically been carried out for *A. thaliana* with Trp and IAA derivatives chlorinated in most possible positions on the indole ring. Finally, the expression patterns of selected genes encoding proteins from the IAA synthesis pathway involving indole-3-acetonitrile (IAN) have been studied.

## 2. Results

### 2.1. Effect of Halogenated Compounds on the Growth of Arabidopsis thaliana Wild Type

**Figure 1:**
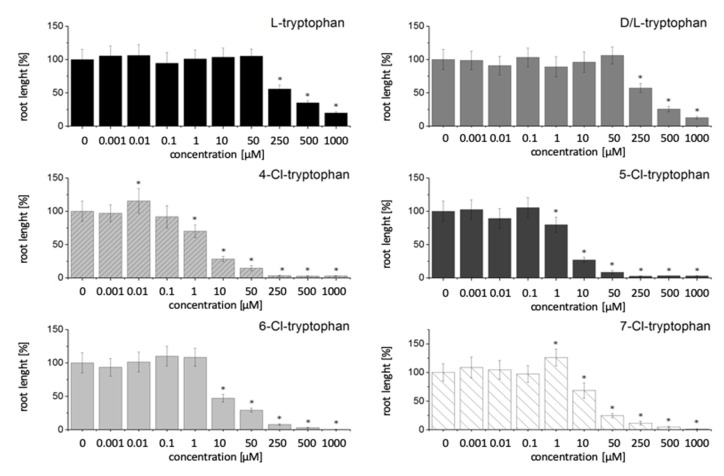
Effect of tryptophan (Trp) and chlorinated Trp derivatives on root length of *A. thaliana* wild type plants. Since all Cl-derivatives were the D/L racemic mixtures, we included the D/L racemate as comparison, but we were also interested to see whether L-Trp had a different effect which was not the case. Concentrations are given in µM. Shown are the relative root lengths 17 days after incubation on different Trp-containing media. The results are calculated based on the control plants without any treatment to make them more comparable between different experiments. Significant differences of *p* < 0.05 in comparison to the control plants are labeled with *. Data are mean values of N > 50 ± SD.

To gain a better understanding of the effect of four different chlorinated Trps and auxins on root growth, these compounds were tested in various concentrations on agar plates with wild type *A. thaliana* plants. Both Trp and IAA derivatives were chlorinated in different positions on the indole ring and compared to non-chlorinated compounds (Figure 1). We choose 17 days of growth on Trp and IAA containing media for our experiments, since this was the time point of analyses for the chlorinated compounds in transgenic lines. Therefore, we wanted to compare the effect of halogenated Trp and IAA derivatives at that specific time point. No significant improvement in growth of the roots was found for any of the tested Trp concentrations (non-chlorinated and chlorinated). At higher concentrations, the growth of the roots was inhibited. Furthermore, the growth inhibition by the chlorinated Trps in comparison to the non-chlorinated compounds starts at significantly lower concentrations, which is an indication of the superior bioactivity or stability of these substances. The lethal dose value (no germination) of non-chlorinated Trps is 5 mM (Figure S1), whereas those for the chlorinated Trps are in the range of 250–500 µM.

**Figure 2:**
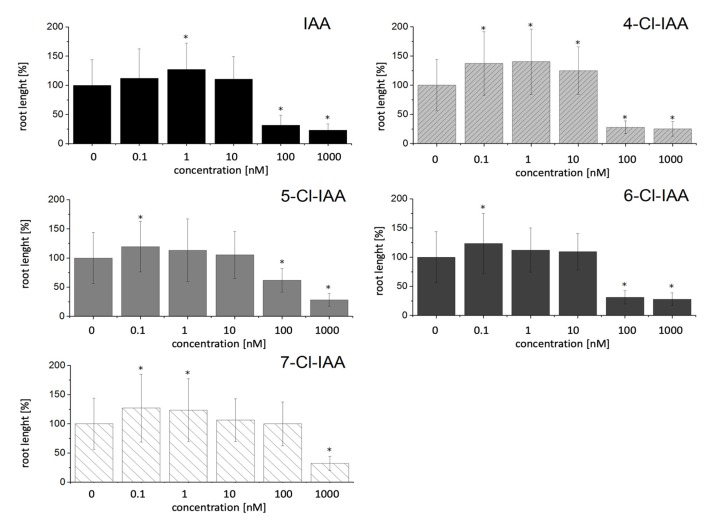
Effect of indole-3-acetic acid (IAA) and Cl-IAA on root length of *A. thaliana* wild type plants. Concentrations are given in nM. Shown are the relative root lengths 17 days after incubation on different IAA- and Cl-IAA-containing media. The results are calculated based on the control plants without any treatment to make them more comparable between different experiments. Significant differences of *p* < 0.05 in comparison to the control plants are labeled with *. Data are mean values of N > 50 ± SD.

For all auxins tested we can show a concentration-dependent optimum curve, albeit with a not very prominent increase in growth in most cases. In lower concentrations up to 1 nM there is a slight increase of the growth of the roots, whereas higher concentrations are inhibitory (Figure 2). At a concentration of 1 nM, the non-chlorinated IAA has a slight optimum with respect of root growth. In comparison to that, very similar to the chlorinated Trp derivatives, the slight growth promotional effects for all chlorinated auxins is found already at a lower concentration of 0.1 nM. At higher concentrations of 100 nM, IAA as well as 4-, 5- and 6-Cl-IAA show a strong growth inhibition, while 7-Cl-IAA is inhibiting root growth only at 1 µM. These results can be viewed as an indication of either a high bioactivity or stability of the chlorinated substances. The relative increase of the root growth for 4-Cl-IAA is somewhat higher compared to IAA and the other Cl-IAA derivatives.

For both groups of Cl-derivatives we found a higher growth inhibition when the 4- and 5-position of the indole moiety was halogenated, while chlorination in the 6- or 7-position resulted in growth inhibition, but to a lesser extent, especially for Trp derivatives. IAA was only less efficient when chlorinated in the 7-position, while all Cl-Trp variants were taken up to the same extent (see Table 1).

### 2.2. Chlorinated Compounds Produced in A. thaliana Wild Type after Incubation with Chlorinated Trp Derivatives

In a previous study we found that the production of 5-Cl-Trp as well as 5-Cl-indole-3-acetonitrile (5-Cl-IAN) is possible in transgenic *A. thaliana* plants [25]. To get a better understanding of the possible chlorinated substances derived from chlorinated Trp we incubated *A. thaliana* wild type plants with five different Trp derivatives, chlorinated in various positions on the indole ring. After 24 h incubation time LC-MS analysis showed the formation of various chlorinated indole compounds (Table 1). 

**Figure 3:**
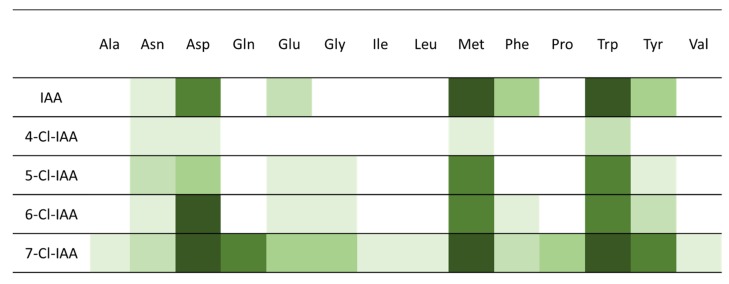
In vitro production of chlorinated IAA amino acid conjugates. Shown are the different produced chlorinated IAA conjugates with AtGH3.3 and different chlorinated IAAs as substrates in an in vitro experiment. Darker green colors = conjugates which are produced in higher amounts; lighter green colors = lower amounts are produced (based on the intensity of the spots of the thin layer chromatography (TLC) plates, Figure S2, and IAA estimates are based on [31]). Ala = alanine, Asn = asparagine, Asp, aspartate, Gln = glutamine, Glu = glutamate, Gly = glycine, Ile = isoleucine, Leu = leucine, Met = methionine, Phe = phenylalanine, Pro = proline, Trp = tryptophan, Tyr = tyrosine, Val = valine.

All chlorinated Trps were taken up into the tissue to the same extent (Table 1). It was also possible to demonstrate the synthesis of Cl-IAN from 4-, 5-, 6- and 7-Cl-Trp. However, the detection of 4-Cl-IAA was not possible, whereas the 5-, 6- and 7-derivatives of IAA were found to have a slight preference for 7-Cl-IAA formation. Similarly, the GH3 protein from *A. thaliana* used a broader range of amino acids when 7-Cl-IAA was the substrate (see Figure 3 and Figure S2). The possibility of forming chlorinated IAA-conjugates has not been shown in other studies. A conjugated form of Cl-IAA, Cl-IAA-Asp, was detectable in all samples after incubation with Cl-Trp derivatives. Especially of interest is the formation of Cl-IAA-Asp, because it is not a storage form in *A. thaliana* and other plant species, rather IAA-Asp is regarded as a precursor for the degradation of the conjugate [29,30]. In addition, the presence of the respective 4-Cl-IAA-Asp derivative could be indirect evidence for the occurrence of 4-Cl-IAA as well.

To get a better insight into the formation of other possible chlorinated conjugates, in vitro investigations were carried out with one GH3 enzyme (AtGH3.3; At2g23170) produced heterologously in *E. coli* and different chlorinated IAA derivatives. We showed that the formation of chlorinated conjugates is possible using AtGH3.3 protein together with all four tested chlorinated auxins and a series of amino acids (Figure 3; the original thin layer chromatography (TLC) plates are shown in the supplement, Figure S2A–D) despite earlier observations by Staswick et al. [31] that 4-Cl-IAA was not significantly conjugated by the IAA conjugating *A. thaliana* GH3 proteins GH3.2, GH3.3, GH3.4, GH3.5, GH3.6 and GH3.17. The formation of the chlorinated conjugates with aspartate, methionine and Trp was very high for all Cl-IAA derivatives. While IAA-Asp is thought to be a substrate in a degradation pathway, IAA-Trp is thought to be an inhibitor of the growth effects of IAA [30]. The conjugate can inhibit IAA-dependent stimulation of lateral roots or eliminate the root inhibition in seedlings which is caused by high IAA concentrations [32]. It is possible that Cl-IAA-Trp has similar effects. Whether the enzymes that catalyze the degradation of IAA-Asp can also metabolize Cl-IAA-Asp has to be demonstrated in the future. Furthermore, other conjugates are formed that are assumed to be storage forms for IAA, e.g., with alanine, glycine or phenylalanine, which are hydrolyzed to yield free IAA upon need [30]. Up to now, it is not known if these IAA amino acid conjugate hydrolases can also cleave the chlorinated conjugates.

### 2.3. Chlorinated Compounds Are Produced in Transgenic A. thaliana Lines but Do Not Alter Growth

**Figure 4:**
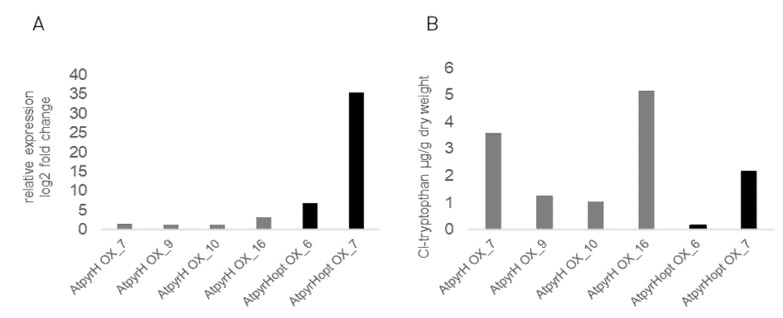
**A**: Analysis of relative gene expression of the corresponding halogenase genes with qRT-PCR. The corresponding reference gene was *AtYLS8*. The normalization was done with the transgenic line with the lowest expression level (expression = 1). **B**: Concentration of chlorinated Trp in transgenic *Arabidopsis thaliana* lines is given in µg/g dry weight. The x-axis describes the individual line names as given in the Materials and Methods Section. For each halogenase gene two optimized (black bars) and four original (grey bars) codon lines were analyzed.

In previously published studies we have shown the synthesis of 5-Cl-Trp, 5-Cl-IAN and 5-Cl-IAA in transgenic *A. thaliana* plants after the stable transformation with the Trp 5-halogenase gene *pyrH*, as well as for the plant codon-optimized gene *pyrHopt* without any co-introduction of a flavin reductase [25]. Since the halogenase genes stem from bacteria, the codon usage might affect the synthesis of the protein in a heterologous host. Therefore, we have included both native and codon-optimized gene constructs also for the two other bacterial halogenase genes used in this study. Similarly to this study with the *pyrH* gene from *Streptomyces rugosporus*, other halogenase genes (Trp 6-halogenase *thdH* from *Streptomyces albogriseolus* and Trp 7-halogenase *prnA* from *Pseudomonas fluorescens*) [25] were commercially synthesized, cloned [25] and expressed under the control of the constitutive CaMV promotor and stably transformed into *A. thaliana* via floral dip [33]. Expression analyses showed that the codon-optimized version of *pyrH* was higher expressed in the two lines chosen compared to four lines of the non-codon-optimized version. Although the different expression level might also be due to positional effects of integration, it could also be the result of a better transcription of the optimized version. However, this difference does not correlate with the levels of Cl-Trp (Figure 4 and Figure S5) and therefore correlation between the codon optimization should also be investigated at the protein level. A higher expression level in the optimized versus natural gene versions was not equally confirmed, since the relative expression of the Trp 6- and 7-halogenase genes show no clear trend in comparison to the original and codon-optimized genes (Figures S3 and S5). We therefore conclude that for the expression and production of three bacterial halogenases in *A. thaliana* no codon optimization is necessary.

In Table 2, the results of various LC-MS analyses from different independent *A. thaliana* lines containing the six different constructs are summarized. We found, in all cases, chlorinated Trp as well as chlorinated IAN. The amount of 5-Cl-Trp varies in the range of 1.7 to 5.15 µg g^−1^ lyophilized tissue (Figure 4). As mentioned earlier, transformants harboring the codon-optimized genes contained not in every independent line more chlorinated Trp than those containing the native gene [25]. The production of Cl-IAA was only detected in some lines harboring *pyrH*, *pyrHopt* and *thdH* (Table 2) and could not be quantified in all lines, since the amounts of the chlorinated products produced were very low. As shown in Table 1, all chlorinated Trp derivatives were converted to Cl-IAA and/or Cl-IAA conjugates. There seems to be a slight preference for 5-Cl-Trp, since there is consistently more of the 5-Cl-derivatives detected, but whether this is due to the better use of the substrates or simply due to more lines not being present or integration specific effects has yet to be determined.

To determine whether *A. thaliana* nitrilase can convert IAN we used nitrilase 2 overexpressing plants (35S::NIT2) [34] and incubated these with 1 mM 5-Cl-Trp. Preliminary data showed that the No-0 wild type had a higher level of 5-Cl-IAN (18–22 µg g fresh weight^−1^) than the overexpressor line (8–12 µg), indicating that the Cl-Trp was further converted via Cl-IAN to other compounds, maybe IAA.

**Figure 5:**
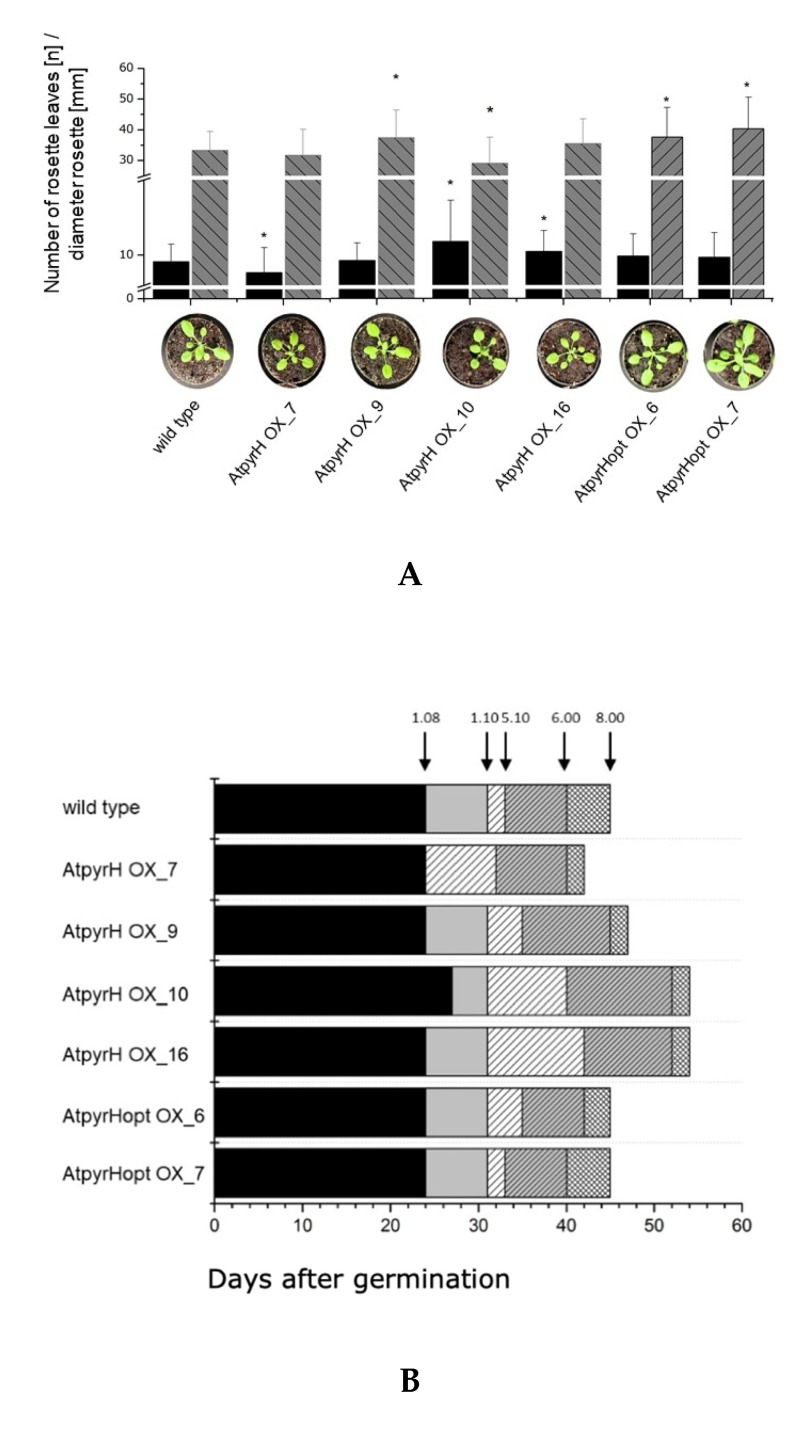
**A**: Numbers of rosette leaves (black histograms) and diameter of the rosettes (grey histograms) of different transgenic *A. thaliana* lines harboring bacterial pyrH in comparison to wild type plants after ca 30 days after germination. Significant differences of *p* < 0.05 in comparison to wild type are labeled with *. Data are mean values of N > 30 ± SD. **B**: Phenotypical analysis according to Boyes et al. [33]. Indicated are the developmental stages of 8 leaves (1.08), 10 leaves (1.10), bud (inflorescence) formation (5.10), flowering (6.00) and pod formation (8.00). The details of the developmental stages are defined in [35]. The days after germination where plants were entering the respective developmental time point are marked by arrows on top of the plot. The respective category was reached when 66% of the examined plants had the same growth characteristic. N > 30. The x-axis (A) and y-axis (B) describe the individual line names as given in the Materials and Methods Section and are the same as in Figure 4. The data for the two other halogenase expressing lines, AtthdH and AtprnA, are shown in the supplement (Figure S4).

The different transgenic lines shown in Figure 4 were examined phenotypically according to different methods, since the expression of a bacterial halogenase gene might result in altered apparent phenotypes due to the different compounds present. First, the rosettes and their leaves on each plant were counted and the rosette diameter was measured. Second, a growth stage analysis according to Boyes et al. [34] was carried out (Figure 5).

Neither the number of rosettes/leaves nor the rosette diameter is significantly altered in comparison to the wild type plants and to other transgenic lines (Figure 5A). When we compared the physiology of the developmental stages, we found some lines which were slower in their development, but there was no correlation between expression of the halogenase gene (Figure 4), chlorinated compounds and development (Figure 5B, Figure S4). This result was similar for the two other groups of halogenase overexpressors; these results are therefore presented in the supplement (Figure S4). Even though there is no correlation between any of the phenotypes and the amount of the respective Cl-Trp derivative present (Figure S5), our initial aim was to show that Cl-Trp and other indole derivatives can be synthesized in *A. thaliana*, which has been achieved for many transgenic lines.

### 2.4. Analysis of Gene Expression from the Trp-Dependent IAA Biosynthetic Pathway in Different Transgenic A. thaliana Lines

**Figure 6:**
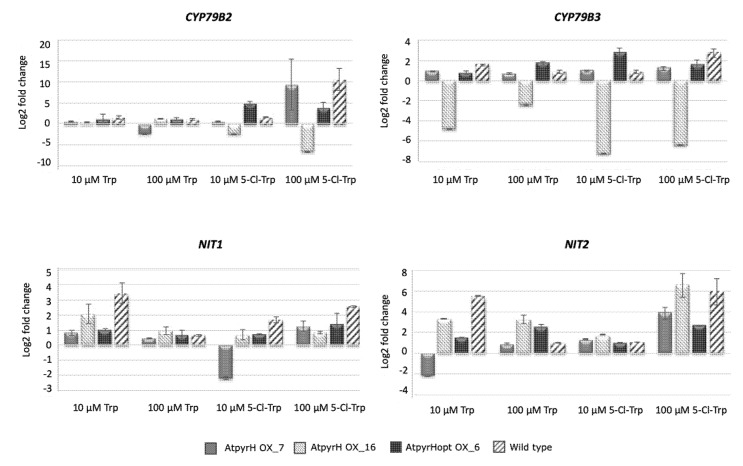
Expression analysis of different transgenic *Arabidopsis thaliana* lines harboring the 5-Cl-Trp halogenase gene *pyrH* and wild type after feeding of Trp and 5-Cl-Trp in different concentrations. Normalization was done to the untreated plants of the respective lines. A fold change higher than 2 in the logarithmic scale means an up-regulation of the respective gene and a fold-change under 2 a down-regulation. CYP79B2, B3 = cytochrome P450 involved in the synthesis of indole-3-acetaldoxime; NIT1, 2 = nitrilase. The individual line names are as given in the Materials and Methods Section.

We have shown previously [25] and in this work that the expression of different halogenase genes in *A. thaliana* resulted in functional enzymes by the formation of Cl-Trp as well as a possible intermediate of the IAA pathway, Cl-IAN, and Cl-IAA. We wanted to analyze whether the presence of Cl-IAA in transgenic plants could have an effect on the expression of genes encoding enzymes of the Trp-dependent IAA synthesis pathway involved in the formation of IAN as an intermediate compared to the wild type. To possibly stimulate the gene expression of the pathway we also added either Trp or 5-Cl-Trp at two different concentrations (Figure 6). Since only the synthesis of chlorinated IAN was observed from Cl-Trp, we concentrated on genes from this specific pathway, namely the genes encoding the two first enzymes of the pathway enabling the formation of indole-3-acetaldoxime from Trp (cytochrome P450 79B2 and B3; CYP79B2, B3) as well as two nitrilase genes (NIT1, NIT2) that encode enzymes involved in the conversion of IAN (and other nitriles) to IAA (and other carboxylic acids). As the involvement of Trp aminotransferase (TAA) proteins in the formation of 4-Cl-IAA in peas has already been demonstrated [27], we were more interested in this alternative crucifer specific pathway. Nevertheless, a more detailed expression analysis including genes from other biosynthetic pathways would be of interest in the future. Since the highest Cl-IAN production was found in the lines overexpressing *pyrH*, we chose a selected set of these transformants for the expression analyses, two overexpressor lines for the native and one for the optimized gene.

The upper part of Figure 6 shows the transcript formation of the two *CYP79B2* and *CYP79B3* genes and the two lower panels display the expression of two nitrilase genes, *NIT1* and *NIT2*. First, we describe the gene expression of wild type in response to the different Trp derivatives at two concentrations. All four gene products were consistently upregulated after treatment with 100 µM 5-Cl-Trp, albeit to a different extent, and both *NIT* transcripts were additionally upregulated by 10 µM Trp. Wild type plants also produced Cl-IAN and Cl-IAA when incubated with different Cl-Trp variants (Table 1), indicating a possible connection to the upregulation of biosynthesis genes (Figure 6). In addition, a 35S::NIT2 overexpressor line indicated faster conversion of Cl-IAN to other metabolites, albeit the latter were not identified yet. These observations point to a role for nitrilase in the conversion of Cl-Trp to Cl-IAA.

Second, we analyze the differences between the transgenic lines and wild type after the treatments. In general, there was no consistent up- or downregulation of any transcripts in the transgenic lines in comparison to the wild type. Thus, the induction/repression of genes in individual transgenic lines seems to be line specific. Nevertheless, there are some interesting observations possible. Both *CYP* genes are downregulated in line AtpyrHOX_16 after all Trp treatments (*CYP79B3*) or only after treatment with 5-Cl-Trp (*CYP79B2*), while *NIT2* expression was mainly upregulated in this line after treatments with Trp and Cl-Trp. However, the second line harboring a non-optimized construct showed a different pattern, namely upregulation of *CYP79B2* and *NIT2* by high concentrations of Cl-Trp, but *CYP79B3* and *NIT1* were either not regulated at all or, in one case, downregulated. In line AtpyrHopt OX_6 there was an upregulation of *CYP79B2* for one and *CYP79B3* for all Cl-Trp treatments, while only *NIT2* expression was upregulated by both Trp derivatives at high concentrations. In conclusion, upregulation of *NIT* transcription in some lines, including wild type, by Trp and Cl-Trp (at different concentrations) could indicate a role for this enzyme in the conversion of high levels of precursor and also, together with the *CYP79B2/B3* genes, in stress response (see Section 3.3.)

## 3. Discussion

### 3.1. Effect of Halogenated Compounds with an Indole Moiety on Growth of A. thaliana Wild Type 

In previous studies with L-tryptophan, hormone-like effects, either through the compound itself or with conversion to IAA, could be demonstrated for root growth by adding different Trp concentrations to *Lactuca sativa*, cotton and maize. At lower concentrations, the growth of the roots increased, while higher concentrations inhibited the growth of the roots [36,37,38]. In our investigations with L-Trp, we could only transiently verify these hormone-like effects on the root growth of *A. thaliana*. At higher concentrations we also found that the growth of the roots was inhibited (Figure 1). Additionally, we investigated the root growth of *A. thaliana* with different chlorinated Trp derivatives, which has not been reported so far. However, these experiments show that there are no highly significant growth increases. For all Cl-Trp compounds it was observed that the inhibition of the root growth starts at a much lower concentration than after the addition of Trp. These results are first indications that the chlorinated compounds have a significantly higher bioactivity compared to the non-chlorinated compounds. Whether the described effects are due to the effect of the substance itself or whether a conversion of Trp into other metabolites such as IAA leads to these effects cannot be conclusively clarified. In future work, auxin biosynthesis mutants should be used to answer this question.

While non-chlorinated auxin-like compounds have been extensively studied, work on halogenated auxins is less well documented. However, the well-studied synthetic 2,4-dichlorophenoxyactic acid with auxinic, but also herbicidal activity, is a dichlorinated compound [39]. In addition, there are a few reports on the use of synthetic halogenated IBA analogs, for example trifluoro-IBA, on the root growth of maize seedlings [40]. For IAA, it is known that the effect on the growth of the plants is a concentration-dependent effect [41]. As already mentioned, 4-Cl-IAA is one of the strongest auxins in terms of different parameters such as elongation growth, protoplast swelling, among others, in maize [3]. Exogenous 4-Cl-IAA can significantly promote the growth of pea pericarp compared to non-chlorinated auxin [6,42]. However, at higher concentrations, as with IAA, there is an inhibitory effect on growth [42]. In addition to the known 4-Cl-IAA, we also tested other chlorinated auxins for their effects on the growth of *Arabidopsis thaliana* wild type roots. In addition to 4-Cl-IAA, we have also examined various other Cl-IAA derivatives in more detail. The optimum curves described in the literature can be determined for all tested chlorinated IAA derivatives, but in our experiments the optimum is not as pronounced and found at significantly lower concentrations. This result is partly consistent with the results from the literature, which speak of a tenfold stronger effect of 4-Cl-IAA [3], whereby the growth of the pea pericarp was promoted only by 4-Cl-IAA, whereas the addition of 5-, 6- and 7-Cl-IAA at the same concentrations shows no growth-promoting effects [42].

In addition to the naturally occurring auxin 4-Cl-IAA, the other Cl-IAA derivatives show the same growth effects in our investigations with *A. thaliana* compared to IAA at lower concentrations. These results once again illustrate the potential of halogenated compounds for plants. Another halogenated auxin, 5-fluor-IAA (5-F-IAA), also has a growth promoting effect on *A. thaliana* [43]. This compound was also used to distinguish between different auxin signaling pathways as it only activates the TIR1 pathway, but 5-F-IAA could not induce the protoplast swelling as it is the case for 4-Cl-IAA [44]. Obviously, the different halogenation positions and/or halogen atoms can induce different physiological responses. Our results suggest rather an effect on the type of halogen than the position of the atom, since *A. thaliana* roots grown on 4- and 5-Cl-IAA behaved similarly (Figure 2). Recent work has shown that at least part of the strong effect of 4-Cl-IAA in peas is due to altered auxin receptor populations of the TIR/AFB family [45]. Furthermore, this effect could be mimicked in *A. thaliana* by showing that root elongation was reduced in a *tir1* mutant and could be restored by expressing genes encoding the pea receptors.

### 3.2. Which Chlorinated Compounds Can Be Produced in A. thaliana Wild Type?

From studies with *Catharanthus roseus* hairy roots, it is known that the introduction of the Trp-7-halogenase gene *rebH* leads to the formation of chlorinated Trp and tryptamine as well as chlorinated indole alkaloids derived from the indole core structure [46,47]. So far nothing is known about whether *A. thaliana* is able to use and metabolize this chlorinated amino acid to build Cl-IAA or other Cl-IAA precursors. The main biosynthesis pathway of IAA in *A. thaliana* is the indole pyruvic acid pathway involving the Trp aminotransferase TAA1 [48]. In peas, three homologs to TAA1 have been isolated (PsTAR1, PsTAR2 and PsTAR3), which all have about 51% homology to TAA1. In in vitro tests with 4-Cl-Trp, it was possible to show that PsTAR1 and PsTAR2 can metabolize 4-chloroindole-3-pyruvate to produce 4-Cl-IAA [27]. Furthermore, deuterium-labeled Trp was injected into the endosperm of pea seeds and it was possible to show the formation of labeled 4-Cl-Trp as well as 4-Cl-IAA [27]. These studies indicate that 4-Cl-IAA is derived from the chlorinated amino acid 4-Cl-Trp in peas [26,27]. In various plants, such as *Medicago truncatula* and *Trifolium repens*, there is evidence that chlorinated Trp is also a precursor for Cl-IAA [16].

In feeding experiments we were able to show not only the uptake of chlorinated Trp but also its metabolism. The conversion of Cl-Trp variants that have the Cl-atom at different positions on the indole ring to the respective chlorinated indole derivatives in *A. thaliana* was demonstrated. This shows that the enzymes involved in Trp-dependent IAA synthesis can use the modified substances and finally form Cl-IAA in *A. thaliana* as well. It was conspicuous that the plants formed a certain IAA conjugate (Cl-IAA-Asp) with all four tested Cl-Trp derivatives as substrates. *A. thaliana* conjugates of IAA and Asp are thought not to be a conjugate for the storage of IAA, but a precursor for degradation [30,49]. One possible explanation for the rapid formation of this conjugate could be the increased bioactivity of 4-Cl-IAA. The plant is taking the degradation route by converting 4-Cl-IAA to its aspartate conjugate rather than forming conjugates that are hydrolysable. Furthermore IAA-Asp plays an important role in abiotic stress and ripening [50,51]. No other chlorinated conjugates could be detected in the samples. Nevertheless, the question arose if further conjugates could possibly be formed by the family of amino acid conjugate synthetases [52] using chlorinated IAA derivatives in vitro. In our enzyme assay, only one synthetase from the GH3 family was tested (GH3.3), but in combination with several amino acids as possible conjugation partners to different chlorinated IAA derivatives it was shown that the enzyme also accepted chlorinated IAA derivatives. In addition to the Cl-IAA-Asp, also already detected in planta (Table 1), further chlorinated conjugates were formed (Figure 3). This is contrary to what was reported by Staswick et al. [31] who could not find activity with the six IAA conjugating enzymes in vitro using 4-Cl-IAA as substrate. Our investigations prove that the enzyme AtGH3.3 is also able to form conjugates with chlorinated IAA variants.

### 3.3. Chlorinated Compounds in A. thaliana and Their Effect on Plant Growth

The optimization of bacterial genes should lead to their better transcription in a plant system and ultimately enable a stronger expression. For other expression systems, it was possible to show that the optimization of genes for the corresponding organism leads to an increased expression and increased formation of enzymes [53,54]. However, we found that the optimization did not necessarily lead to an increased expression when comparing the expression data between the optimized and original halogenase genes (Figure 4). Only for the Trp-5-halogenase genes *pyrH* and *pyrHopt* we were able to demonstrate such a difference in favor of the transcription of the latter. For all other constructs and the resulting gene expression values, only individual differences between individual lines were determined. These disparities within a set of lines harboring the same bacterial halogenase gene could have different reasons. One could be the integration of the halogenase genes into different sites within the genome of *A. thaliana*. Furthermore, an increased expression does not necessarily lead to an increased accumulation of Cl-Trp. In our studies it was not possible to analyze the amounts of enzymes present in the individual lines, so we can only draw conclusions from the formation of the chlorinated product Trp. In *A. thaliana*, Trp is the precursor of many different metabolites such as IAN, IAA [55], camalexin [56] and indole glucosinolates [57]. To what extent Cl-Trp was metabolized to other substances or degraded we cannot conclude from our experiments, since the other putative metabolites were most likely below the detection limit, especially since they are regarded as stress metabolites and the plants for metabolite measurements were grown under sterile conditions.

For different bacterial halogenases (PyrH, ThdH, PrnA) it was shown in vitro that these enzymes are enabled to halogenate IAA directly [25]. However, the activity of halogenases towards IAA as substrate is much lower than with Trp. In peas, the biosynthesis of 4-Cl-IAA is postulated as starting from Cl-Trp. Three TAA1 homologous were isolated, which are involved in the formation of 4-Cl-IAA by the formation of 4-chlorindole-3-pyruvate [27]. However, the enzymes involved in the synthesis of 4-Cl-Trp have not yet been identified [16,27].

Except for Cl-Trp and Cl-IAN, no other chlorinated intermediates have been found in transgenic plants. This is mainly due to the low concentrations of the chlorinated substances formed. We found not more than 5 µg·g^−1^ of Cl-Trp and about 0.2 µg·g^−1^ Cl-IAN, each based on the dry weight. If a conversion from Cl-IAN to Cl-IAA had taken place, it could be possible that this was not detected due to the low concentrations. As the possibility exists that small amounts of IAA and Cl-IAA are degraded under light, we used yellow protective covers for these experiments to minimize that effect. As shown before, IAA and Cl-IAA were stable in solid and liquid medium over a period of 17 days, as about 80% of the compounds were still detectable [25]. Furthermore, there is a possibility that the efficiency of the corresponding enzymes which are involved in the formation to Cl-IAA is less to the chlorinated intermediates, such like Cl-IAN or Cl-Trp. It is known that IAN can be converted to active IAA by a group of nitrilases (mainly NIT1 and NIT2 isoforms) [34,58,59]. Up to now, nothing is known about alterations in their efficiency in *A. thaliana* towards modified substrates. Preliminary experiments point to the possible conversion of 5-Cl-Trp to downstream metabolites to a higher extent than wild type by a nitrilase overexpressing line (35S::NIT2; [34]). So far, this is rather indirect, since we found a reduction of Cl-IAN but have not detected Cl-IAA.

When a substrate of a biosynthetic pathway is added, this usually results in higher levels of product, but we also wanted to know whether chlorinated compounds affect the expression of genes encoding enzymes for the *A. thaliana* specific pathway via IAN, via which we detected the chlorinated compounds. We therefore analyzed the expression of two cytochrome P450-dependent genes encoding enzymes of the first committed step to indole glucosinolates/IAA (*CYP79B2*/*B3*) [60] and also the transcription of the two major nitrilase genes (*NIT1*/*2*) encoding the enzymes responsible for the formation of IAA from IAN [58,59]. The idea was to investigate, first, whether an endogenous increase in Cl-metabolites would result in an alteration of transcripts of the pathway and second, whether the expression of the genes would be altered after incubation with Trp or the respective Cl-Trp. The experiment was done with a transgenic line harboring the 5-Cl-Trp halogenase gene *pyrH* and a plant expression-optimized version of the same gene. Consequently, the incubation was done with the 5-Cl derivative. However, only line-dependent alterations in gene expression were detected. Therefore, we cannot rule out that some of the effects observed could be due to the known stress response of some pathway genes resulting in altered gene expression [59], although this seems not likely, as for these experiments the plants were grown under sterile conditions in plastic bowls. Nevertheless, an increase in Cl-IAN or Cl-IAA might be possible in plants with higher expression levels of such genes as a starting material for the transformation with halogenase genes in the future.

## 4. Materials and Methods

### 4.1. Plant Material and Growth Conditions

*Arabidopsis thaliana* (L.) HEYNH. ecotype Col-0 was used as the wild-type controls as well as for generating transgenic plants overexpressing the different halogenase genes (*pyrH*, *thdH*, *prnA*,) resulting in lines AtpyrH, AtpyrHopt, AtthdH, AtthdHopt, AtprnA, AtprnAopt. To generate the transgenic plants the original and optimized (opt) genes (optimization and gene synthesis for both variants was done by Life Technologies, Regensburg, Germany) were amplified using PCR with specific primers (Tables S1–S6) and inserted via the Gateway cloning technology into the pMDC32 vector [61]. In this plant expression vector, the halogenases are under the control of the constitutive cauliflower mosaic virus 35S promotor (CaMV). The final constructs were transformed into *Agrobacterium tumefaciens* GV3101 strain and afterwards using floral dip infiltration [33] into the *A. thaliana* wild type plants. The selection of the transgenic plants was done with hygromycin resistance. To check that the genes of interest are present in the plants as well as expressed, DNA and RNA were extracted and PCR as well as RT-PCR were performed using genomic and cDNA as templates. *A. thaliana* ecotype No-0 was used in comparison with a nitrilase 2 overexpressing line (35S::NIT2) [34].

The *A. thaliana* plants were grown under greenhouse conditions (16/8 day/night, 23 °C/18 °C) or sterile in petri dishes and liquid cultures in plastic cups (Wächter & Co. GmbH Kunstoffwarenfabrik, Leopoldshöhe, Germany). The sterile cultivation for selection (with 50 µg hygromycin), the growth tests and the feeding experiments were performed with standard MS medium containing 1% sucrose, pH 5.8. For the petri dish tests 1.5% phyto-agar was added together with the appropriate concentrations of chlorinated Trp or auxin derivatives (from commercial sources: Biosynth AG, Thal, Switzerland; Fluorochem, Hadfield, UK; Matex, Singapore, Singapore). The seeds were sterilized by treating them with 75% ethanol for 5 min, followed by 15 min in a sodium hypochlorite solution and four wash steps in sterile distilled water. The seeds were stratified in the dark at 4 °C for two days. The incubation was performed in growth chambers at 23/18 °C under a 16/8 h light/dark period for 17 days or in the greenhouse on a shaker (60 rpm). We chose this time point because (i) the same was used for the other analyses where we needed to collect enough material and (ii) because prominent differences were only found at such later time points. To protect IAA and Cl-IAA from degradation, yellow plastic shields were used [25].

For the routine greenhouse cultivation and the phenotype analysis the plants were placed after 10 days on the selection media in pots with soil and sand (3:1). The phenotypic studies of the plants were done following Boyes et al. [35]. For every line more than 30 plants were observed over the time and various stages were considered and the specific developmental stage noted.

### 4.2. RNA Extraction and qRT-PC Analysis

The plants were incubated in liquid MS culture under sterile conditions for 14 days and treated for 24 h in the corresponding Trp- or 5-Cl-Trp-containing medium. The plant material was mortared with liquid nitrogen and RNAzol (Sigma-Aldrich Chemie GmbH, Munich, Germany) directly after harvesting. After being mortared, the plant material samples were stored at −80°C until RNA extraction. The RNA extraction was performed following the manufacturer instructions. In addition to this a DNA removal step was added, to get DNA-free RNA. The digestion of the DNA was performed with DNase I (Life Technologies, Carlsbad, California, United States) and 1–20 µg RNA. For the quantitative real-time PCR (qRT-PCR) the cDNA was synthesized with Maxima First Strand Reverse Transcriptase (Life Technologies, Carlsbad, California, United States) following the manufacturer information with 1 µg RNA. The SensiFAST SYBR No-Rox Kit by Bioline was used for qPCR as described by the manufacturer. All reactions were performed as triplicates with the following conditions: 95 °C for 5 min, 40 cycles each at 95 °C for 20 s and 62 °C for 20 s, and 72 °C for 20 s. After the cycles a melting curve was performed to confirm that the correct product was synthesized. Specific primers used for qRT-PCR are shown in Supplementary Tables S1–S6. Relative gene expression was quantified using the expression of *AtYLS8* (AT5G08290.2) as control.

### 4.3. UPLC-QqQ-MS Analysis

The analysis of possible chlorinated products in the plant extracts of the transgenic *A. thaliana* plants, as well as the wild type plants which were incubated with the chlorinated Trp derivatives, were done as described in Patallo et al. [25]. In both cases the material was harvested and freeze dried. The extraction was done with 100% methanol (1 mL/50 mg freeze dried material). UPLC-MS analysis was performed as reported in Patallo et al. [25]. Multiple reaction monitoring (MRM) signals for chlorinated Trps (239.2 > 222.2; CE 12 eV), chlorinated IAAs (210.05 > 164.05; CE 18 eV), IAA (176.05 > 130.1; CE 10 eV) and IAN (157.10 > 130.1; CE 10 eV) were optimized using authentic standards. MRM signals for IAA conjugates were set up based on the work of Novák et al. [62], and the collision energy for the fragmentation these compounds (CE 18 eV) was optimized using an authentic standard of IAA-Asp (Carbosynth, Compton, UK). The linear dynamic range was determined for chlorinated Trps (0.025–1 μg/mL; R^2^ = 0.9972), chlorinated IAAs (0.01–1 μg/mL; R^2^ = 0.9989), IAA (0.01–1 μg/mL; R^2^ = 0.9972) and IAN (0.01–1 μg/mL; R^2^ = 0.9950) and the curves were used for quantification. Since standard compounds for each of the IAA conjugates were not available, we used the normalized peak areas as a relative measure of the abundance of the compounds.

### 4.4. Enzyme Assay for GH3 Activity and TLC Analysis

Whether the formation of chlorinated IAA conjugates with the enzyme AtGH3.3 is possible was investigated with an enzyme test. The *GH3.3* gene from *A. thaliana* was PCR-amplified from cDNA essentially as described for the cloning of *Physcomitrella patens* GH3 genes and cloned into the same expression vector as described in [63]. The vector including the GH3.3 cDNA was expressed in *E. coli* BL21 cells, and for the purification of the enzyme we used an IPTG inducing expression plasmid with a glutathione tag. For the purification we applied the protein onto a glutathione-sepharose matrix. An overnight culture of the cells (250 mL LB medium with 2% glucose, ampicillin 100 µg/mL, chloramphenicol 50 µg/mL, tetracycline 10 µg/mL, 37 °C, 180 rpm) was centrifuged for 10 min (3000× *g*, 4 °C). The pellet was resuspended in 250mL standard LB media, but without glucose (ampicillin 100 µg/mL, chloramphenicol 50 µg/mL, tetracycline 10 µg/mL, 37 °C, 180 rpm), and IPTG (1 mM) was added. The culture was incubated for 3–4 h (22 °C, 180 rpm). The cells were centrifuged again (10 min, 3000× *g*, 4 °C) and washed with 2 ml TB buffer. The resulting pellet was frozen in liquid nitrogen. The purification with the glutathione-sepharose matrix was carried out according to the manufacturer’s specifications (GE Healthcare, Chicago, IL, United States). The elution of the GST-fusion protein was done for 10 min with 30 µl elution buffer (GB buffer). The concentration of the eluted protein was analyzed with SDS page and BSA standards. For the enzyme assay, IAA, 4-Cl-IAA, 5-Cl-IAA, 6-Cl-IAA and 7-Cl-IAA in a concentration of 1mM were used as substrates. The reaction mixture consisted of reaction buffer 1×, ATP 3 mM, DTT 1 mM, amino acids 1 mM and 2.5 µg purified protein. The reaction mixture was incubated overnight at 25 °C, frozen in liquid nitrogen and stored at −10 °C until analysis.

The formation of possible chlorinated IAA conjugates from the enzyme assay was subsequently analyzed in more detail using TLC. The free and conjugated IAA were separated for 15 min on Silicia Gel F254 plates in the solvent mixture of chloroform:ethyl acetate:formic acid (35:55:10). The focus was not on the quantification of the samples, but on the formation of possible conjugates with Cl-IAA. Nevertheless, uniform amounts of starting material were applied. The conjugate formation was visualized with Van Urk-Salkovski reagent. The dried plates were sprayed with the reagent mixture and incubated for 10 min at 60 °C [64].

### 4.5. Statistical Analysis

The ANOVA analysis was done using SPSS software (ver. 16.0; SPSS Inc., Chicago, IL, USA). Statistically significant differences were indicated at *p* < 0.05 or *p* < 0.01 level.

## 5. Conclusions

While the native enzyme converting Trp to the Cl-derivative in pea until today is not known, we have successfully shown that heterologous expression of different bacterial halogenase genes in *A. thaliana* is possible and that this can result in the regioselective formation of chlorinated Trp derivatives depending on the type of halogenase produced. In addition to Cl-Trp, we have shown that the synthesis of metabolites from the IAA biosynthetic pathway, namely Cl-IAN, and the auxin itself, Cl-IAA, starting from Cl-Trp in *A. thaliana* is possible. In addition, an increased efficacy of chlorinated compounds (both Trp and IAA) on the growth of wild type plants has been demonstrated. Formation of halogenated compounds, however, did not lead to phenotypic changes in the transgenic plants. Our results provide the basis to use these strategies in the future in other *A. thaliana* lines with altered spectra of metabolites of the pathway to produce higher amounts of the more bioactive chlorinated auxins or other compounds derived from the Trp moiety, i.e., camalexin or indole glucsoinolates.

## Figures and Tables

**Table 1:** Production of different chlorinated indole compounds after feeding chlorinated Trp to wild type *A. thaliana* plants. Plants were grown for 21 days in liquid medium and 100 µM of Trp derivatives was added. IAN = indole-3-acetonitrile, IAA = indole-3-acetic acid, IAA-Asp = IAA conjugate with aspartate, Trp = tryptophan. Concentration ranges are given as µg g dry weight^-1^. For Cl-IAA-Asp the peak could be detected but was below the quantification level (+ present; ++ higher levels present). 4-Cl-IAA could not be detected in the sample (−).

Metabolite	Control	Trp	4-Cl-Trp	5-Cl-Trp	6-Cl-Trp	7-Cl-Trp
Cl-Trp	−	−	1.5–4.0	2.0–2.5	1.3–1.8	1.7–2.0
Cl-IAN	−	−	1.0–6.0	1.0–2.0	0.3–0.6	0.4–0.7
Cl-IAA	−	−	−	0.25–0.4	0.07–0.3	0.7–0.9
Cl-IAA-Asp	−	−	+	++	+	++

**Table 2:** Analysis of chlorinated compounds in different transgenic *Arabidopsis thaliana* lines. The values (µg/g dry weight) are approximates, as reproducible results were not obtained in all samples, and given as a range of lowest to highest detected value over all lines where the compound was detected. In samples indicated with (+) a peak was identified but that was below the limit for quantification. (−) indicates below detection limit.

Line	Cl-Trp	Cl-IAN	Cl-IAA
AtpyrH	1.5–5.5	~0.2	0.07–0.09
AtpyrHopt^1^	0.2–2.0	~0.1	0.07–0.08
AtthdH	~1.1	0.04–0.13	+
AtthdHopt^1^	+	~0.01	−
AtprnA	+	+	−
AtprnAopt^1^	+	+	−

^1^ for plants optimized gene version.

## Supplementary Materials

Supplementary materials can be found at https://www.mdpi.com/1422-0067/21/7/2567/s1.

## Abbreviations

4-Cl-IAA	4-chloro-indole-3-acetic acid
5-Cl-IAA	5-chloro-indole-3-acetic acid
6-Cl-IAA	6-chloro-indole-3-acetic acid
7-Cl-IAA	7-chloro-indole-3-acetic acid
4-Cl-IAN	4-chloro-indole-3-acetonitrile
5-Cl-IAN	5-chloro-indole-3-acetonitrile
6-Cl-IAN	6-chloro-indole-3-acetonitrile
7-Cl-IAN	7-chloro-indole-3-acetonitrile
4-Cl-Trp	4-chloro-indole -tryptophan
5-Cl-Trp	5-chloro-indole -tryptophan
6-Cl-Trp	6-chloro-indole -tryptophan
7-Cl-Trp	7-chloro-indole -tryptophan
5-F-IAA	5-fluoro-indole-3-acetic acid
CYP79B2	cytochrome P450 79B2
CYP79B3	cytochrome P450 79B3
EB	Ethidium bromide
GH3	Gretchen Hagen 3 enzyme
GST	Glutathione-S transferase
IAA	indole-3-acetic acid
IAA-Asp	IAA conjugate with aspartate
IAA-Trp	IAA conjugate with tryptophan
IAN	Indole-3-acetonitrile
IBA	indole-3-butyric acid
IPTG	isopropyl-β-d-thiogalactopyranoside
LB	Luria Bertani medium
MS	Murashige & Skoog medium
NIT1	nitrilase 1
NIT2	nitrilase 2
TLC	thin layer chromatography
Trp	tryptophan
