# Supplementary material for: Chlorinated Auxins—How Does Arabidopsis Thaliana Deal with Them?
Source: Int J Mol Sci. 2020 Apr 7;21(7):2567. doi: 10.3390/ijms21072567 (PMC7177246; doi:10.3390/ijms21072567)
Supplement: Supplementary file 1 [file ijms-21-02567-s001.pdf]

Supplement

Figures

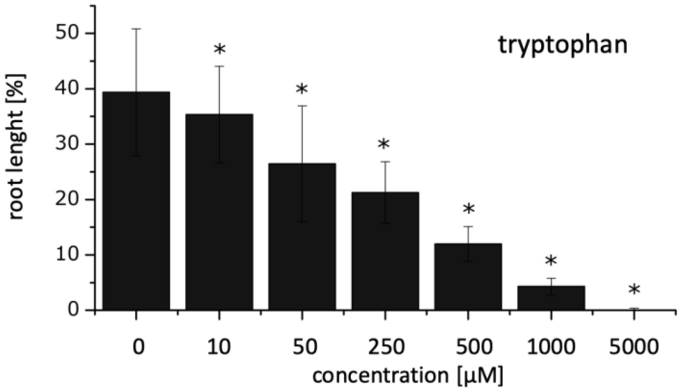

Figure 1. Determination of the tryptophan concentration where the seeds did not germinate any more. Shown are the relative root lengths 17 days after incubation on different tryptophan containing media. The results are calculated based on the control plants without any treatment to make them better comparable between different experiments. Significant differences of $p<0.05$ in comparison to the control plants are labeled with *. Data are mean values of N> 50 ± SD. The data are from the same experiment as in Fig. 1.

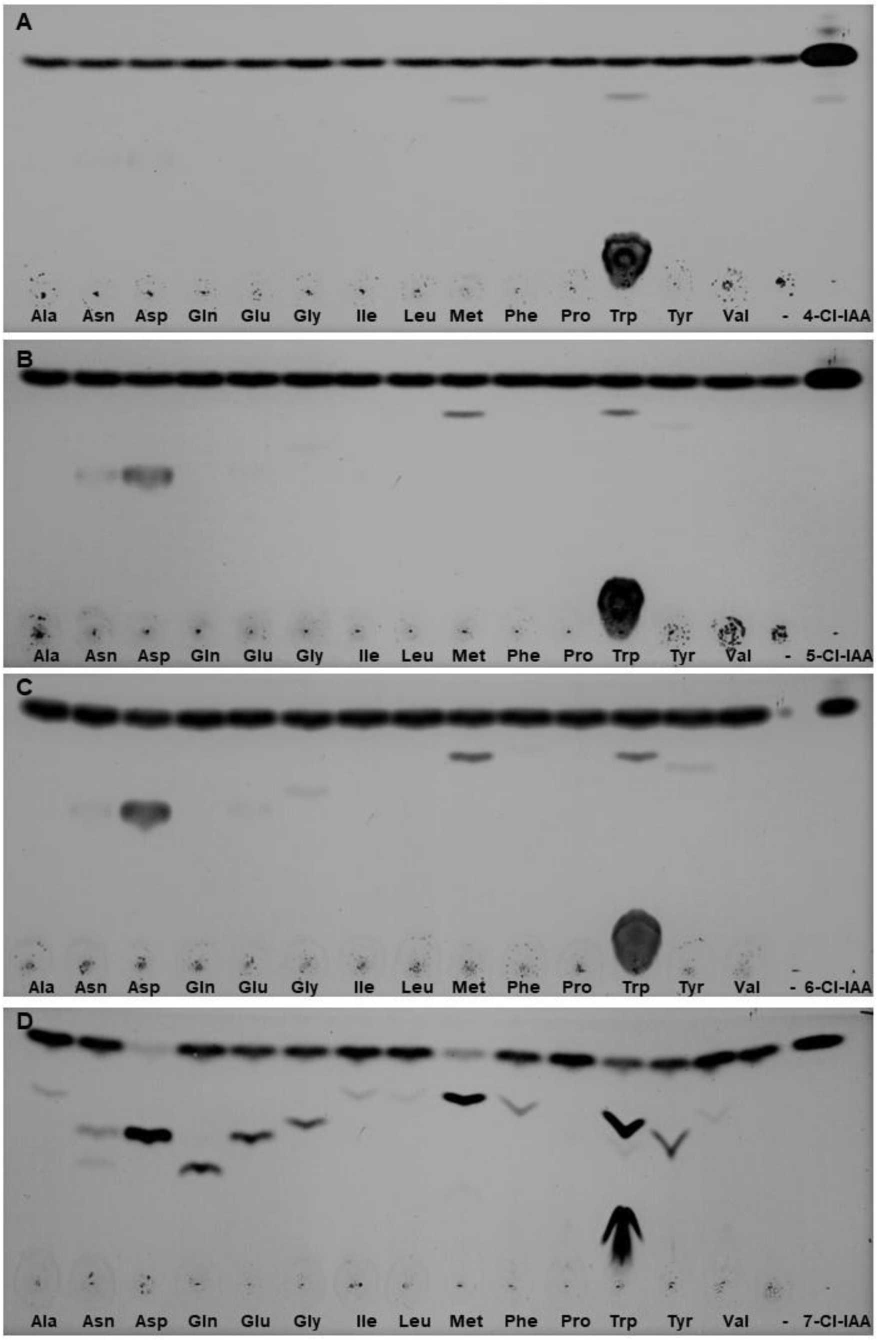

Figure 2. TLC plates of the I*n vitro* production of chlorinated IAA amino acid conjugates. Shown are the different produced chlorinated IAA-conjugates with AtGH3.3 and different chlorinated IAAs as substrates in an in vitro experiment. **A**: 4-Cl-IAA, **B**: 5-Cl-IAA, **C**: 6-Cl-IAA, **D**: 7-Cl-IAA. The upper bands correspond to the unconjugated chlorinated auxin (standards are on the right lane), the con jugates are the bands additionally formed. For Trp, the amino acid itself gives a reaction with the reagent, so three spots are visible.

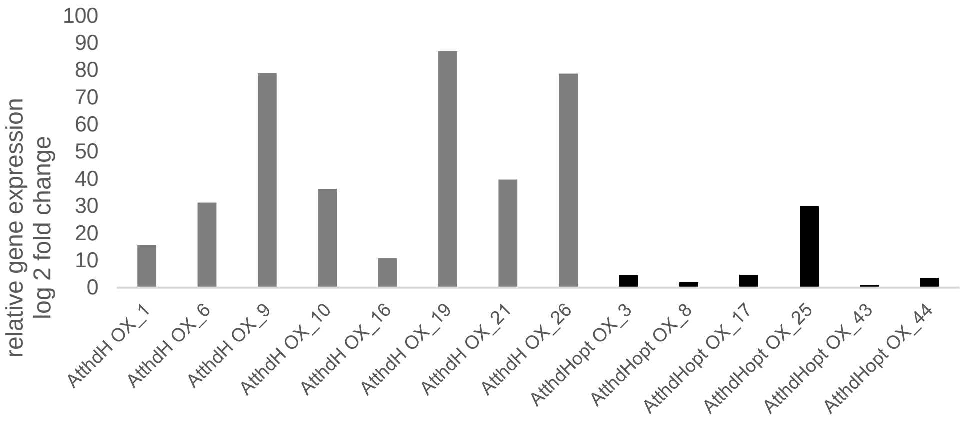

(**A**)

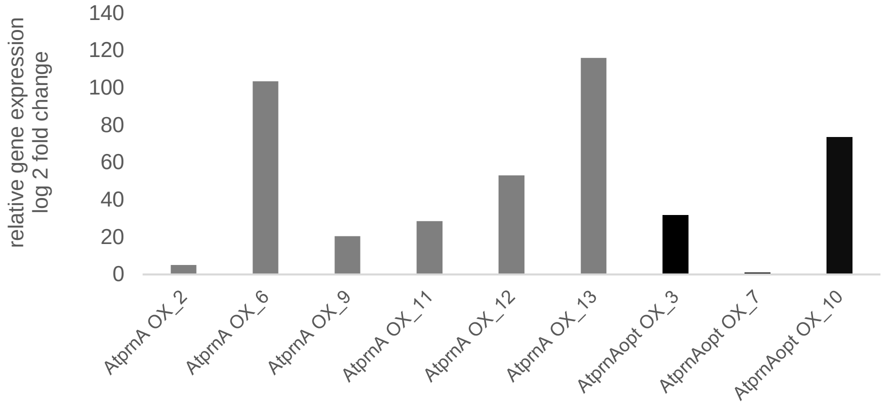

(**B**)

Figure 3. qRT-PCR analysis of the relative gene expression of different *A.thaliana* lines which are expressing the tryptophan 6-halogenase (**A**) and the tryptophan 7-halogenase (**B**) genes. The corresponding reference gene was *AtYLS8*. The normalization was done with the line transgenic with the lowest expression level (expression =1). The x-axis describes the individual line names as given in the Materials and methods section.

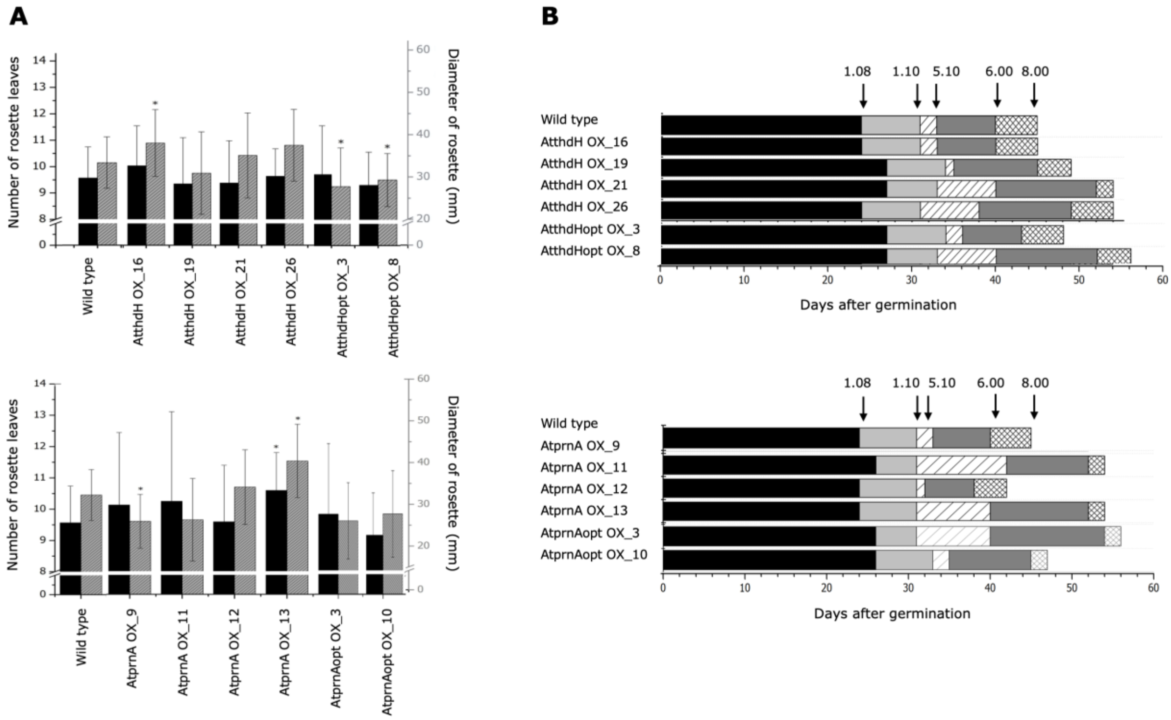

Figure 4. A: Numbers of rosette leaves (black histograms) and diameter of the rosettes (grey histograms) of different transgenic *Arabidopsis thaliana* lines in comparison to wild type plants after ca 30 days after germination. Significant differences of $p<0.05$ in comparison to wild type are labeled with *. N > 30. B: Phenotypical analysis according to Boyes et al. [35]. Indicated are the developmental stages of 8 leaves (1.08), 10 leaves (1.10), bud formation (5.10), flowering (6.00) and pod formation (8.00). The days after germination where plants were entering the respective developmental time point are marked by arrows. The respective category was reached when 66% of the examined plants had the same growth characteristic. N > 30. The x-axis (**A**) and y-axis (**B**) describes the individual line names of different *A. thaliana* lines which are expressing the tryptophan 6-halogenase (*thdH*) and the tryptophan 7-halogenase (*prnA*) genes as given in the Materials and methods section and are the same as in Figure S2.

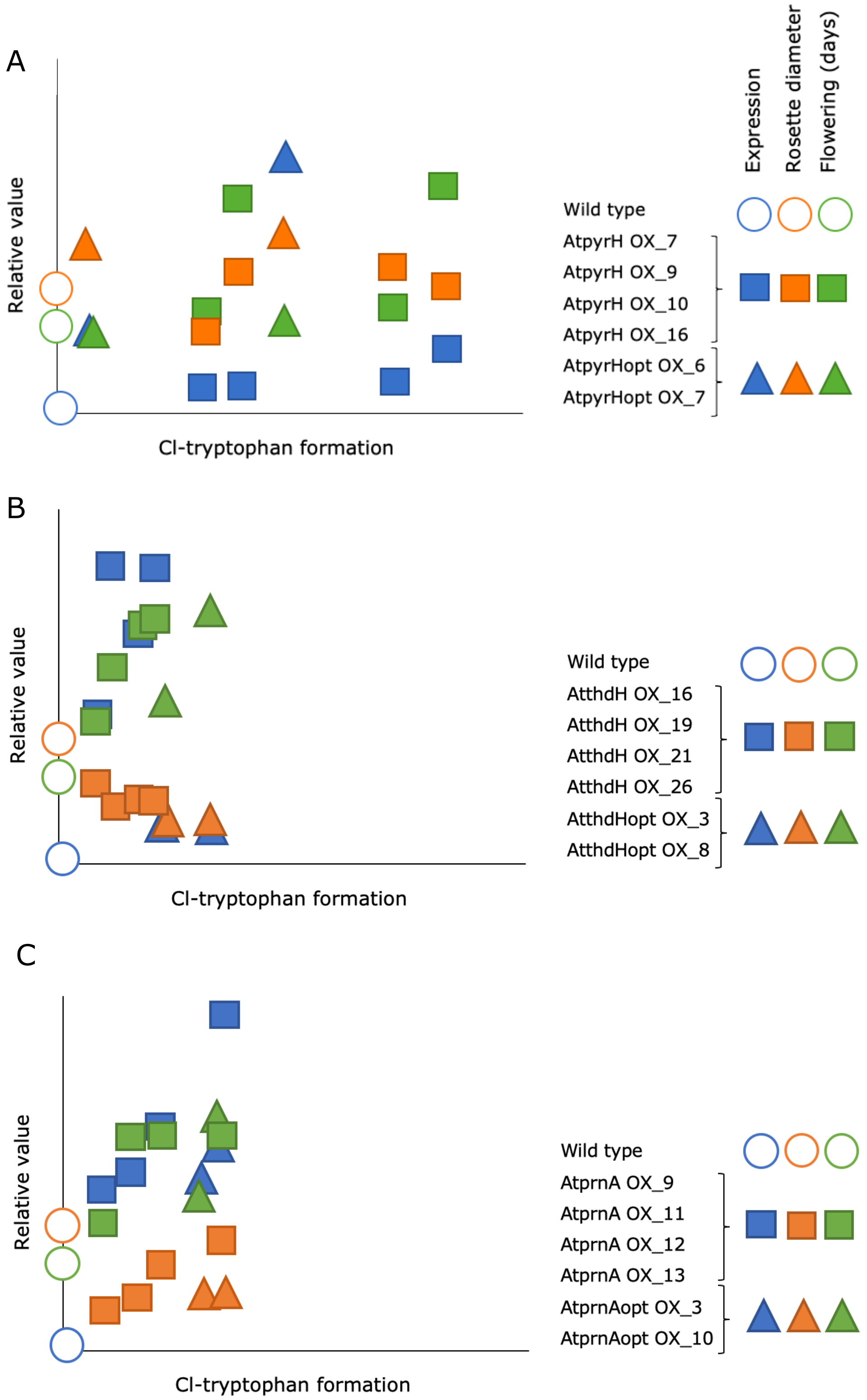

Figure S5. Correlation between halogenase expression (blue), rosette diameter (orange), days to flowering (green) and synthesis of 5-Cl-tryptophan for four lines of pyrH expressing *A. thaliana* plants and two lines with codon optimized construct (**A**). The expression of the halogenase gene resulted always in the formation of Cl-tryptophan, but there was neither a correlation in the amount synthesized nor in phenotypes. For the two other halogenase genes expressed in A. thaliana, thdH (**B**) and prnA (**C**) there was also no correlation found between phenotypes, transcription and Cl-Trp production (see also Figures 4, 5, S2, S3).

Table 1. Oligonucleotides for the tryptophan 5-halogenase.

Primer name	Sequence	Used for
Trp5-H-fw	ATGTATCCGATCTGTGGTGATCGTGG	Amplification of the total length of the gene; gene expression
Trp5-H-rev	TCATTGGTATGCTGGCGAGGTACTCG	
AW-Sr-Trp5-fw	ATGTGGAGCCACCCGCAGTTCGAAAAAATCCG ATCTGTGGTGA	Amplification of PCR products with tags
AW-Sr-Trp5-rv	TCAGTGGTGGTGGTGGTGGTGTTGGATGCTGGC GAGG	
Trp-5-attB-fw	GGGGACAAGTTTGTACAAAAAAGCAGGCTCC ATGTGGAGCCAC CCGCAGTTCG	Amplification of PCR products with att-sites for the creation of the pENTRY-Plasmides
Trp-5-attB-rv	GGGGACCACTTTGTACAAGAAAGCTGGGTCTC AGTGGTGGTGG TGGTGGTGTTGG	
Trp-5-attB-fw2	AGGCTCCATGTGGAGCCACCCGCAGT TCGAAAAAATCCG	
Trp-5-attB-rv2	TGGGTCTCAGTGGTGGTGGTGGTGGTGTTGGAT GCTGG	
qTrp5-fw	CTCGTCCTCCACTACAAGGG	qPCR analysis
qTrp5-rv	GAATAGGTCTCGAAGCCGTG	

Table 2. Oligonucleotides for the optimized tryptophan 5-halogenase.

Primer name	Sequence	Used for
Trp5O-fw	ATGAGAGGGAATGGCTTCCT	Amplification of the total length of the gene; gene expression
Trp5O-rv	GATTCCGAGTCCAAGGTTCA	
Trp5O-Sequ1	GGTGATCTCTTCGTTGATTGC	
Trp5O-Sequ2	TCTATGAGTGAGGTAGCAAGC	
Trp5O-Sequ3	TTCATCCAGCACGCTATCGAG	
Trp5O-Sequ4	CTTCTATCTCCCACAGCGAGC	

Table S3. Oligonucleotides for the tryptophan 6-halogenase

Primer name	Sequence	Used for
AW-SA-Trp6H-fw	ATGTGGAGCCACCCGCAGTTCGAAAAAGACA ATCGAATCAAGA	Amplification of the total length of the gene; gene expression
AW-SA-Trp6H-rv	TCAGTGGTGGTGGTGGTGGTGCGACGCACCGT GCAAC	
Trp6-att-fw	GGGGACAAGTTTGTACAAAAAAGCAGGCTCC ATGTGGAGCCACCCGCAGTTCG	Amplification of PCR products with att-sites for the creation of the pENTRY-Plasmides
Trp6-att-rv	GGGGACCACTTTGTACAAGAAAGCTGGGTCTC AGTGGTGGTGGTGGTGG	
qTrp6/6O-fw	TCCAGGCGCACTACTACTTC	qPCR-Analysen für AtthdH und AtthdHopt
qTrp6/6O-rv	AGTAGTAGCTGCCGTTGGTC	

Table S4. Oligonucleotides for the optimized tryptophan 6-halogenase

Primer name	Sequence	Used for
Trp6O-fw	CCCTGAAGAGGAATGGATGA	Amplification of the total length of the gene; gene expression
Trp6O-rv	TCACATCAGCGAACAAAAGC	
Trp6O-Sequ1	CAGTCTCACCCTGAAGTCTC	
Trp6O-Sequ2	CCAAGCATAGGGATCTTCCA	
Trp6O-Sequ3	GGGAAGAAACAGAAGGGCTTG	

Table S5. Oligonucleotides for the tryptophan 7-halogenase

Primer name	Sequence	Used for
AW-Pf-Trp7H-fw	ATGTGGAGCCACCCGCAGTTCGAAAAAAACA AGCCAATCAAGA	Amplification of PCR products with tags
AW-Pf-Trp7H-rev	CTAGTGGTGGTGGTGGTGGTGCTGGCGTTCCTG CGCC	
Trp7H-att-fw	GGGGACAAGTTTGTACAAAAAAGCAGGCTCC ATGTGGAGCCACCCGCAG	Amplification of PCR products with att-sites for the creation of the pENTRY-Plasmides
Trp7H-att-rv	GGGGACCACTTTGTACAAGAAAGCTGGGTCTC AGTGGTGGTGGTGGTGG	
qTrp7/7O-fw	AGCAGCTCAACCAGATCAAC	qPCR analysis
qTrp7/7O-rv	CGTAGTCGAAGGTCTCGTAG	

Table S6. Oligonucleotides for the optimized tryptophan 7-halogenase

Primer name	Sequence	Used for
Trp7O-fw	CCAGCAGCAGGTTAACATCA	Amplification of the total length of the gene; gene expression
Trp7O-rv	TCTGAGCCTCTCAGCCTCTC	
Trp7O-Sequ1	TCAGGAGACTTCCTCCAATTC	
Trp7O-Sequ2	TCCAGGTCCATCCAGAGTTC	
Trp7O-Sequ3	TCAATCGGACTCTCTTCATGC	
